# Extra Supplement.—The Nursing Mirror

**Published:** 1894-03-31

**Authors:** 


### "The Hospital" Nursing Mirror.

Being the Extra Nursing Supplement of "The Hospital" Newspaper.

[Contributions for this Supplement should be addressed to the Editor, The Hospital, 428, Strand, London, W.C., and should have the word "Nursing" plainly written in left-hand top corner of the envelope.]

### News from the Nursing World.

### SAVED—NOT WASTED.

"Did she belong to the Pension Fund?" was asked of the friends of a nurse recently laid to rest. "Oh, yes," was the reply, "she joined a good while back." "What a pity," said the first speaker; "it seems a regular waste of money, now she has died before her pension was due." "Not at all," returned the friend of the deceased; "it was a great comfort to her to know that her policy would not only cover the funeral expenses, but leave a surplus to be divided amongst several poor pensioners, by whom the loss of their friend is bitterly felt."

### INFANTS' NOURISHMENT AGAIN.

"And what *should* I give my baby to eat," said a mother to a Health Lecturer the other day after listening to a discourse on the wickedness of trying to bring infants up on boiled bread, soaked biscuits, or "a little of what we has ourselves." The lecturer spent half an hour in trying to persuade the mother that milk was the only proper and natural food for infants, but finding her still unconvinced she at length said, "Well, ask the doctor yourself if I am not right." "The doctor," replied the woman in accents of the greatest scorn, "the doctor! Why he don't know no more about babies than you do." The lecturer took refuge in silence, feeling there was small hope of the poor little baby ever escaping from the mixed diet prescribed for it by its mother.

### WARLIKE WOMEN.

So far as the aims of the proposed Women's Volunteer Medical Staff Corps are at present plain to us, they are certainly somewhat ambitious ones. Apparently the ladies not only aspire themselves to undertake the transfer of wounded soldiers on stretchers, but the shoeing of horses, repairing of harness, &c., are to be amongst their duties. The Secretary says: "We shall have to be the reverse of superficial," a doctrine which will impress itself very firmly on the flesh as well as minds of the damsels who attempt to carry bulky veterans. We are glad to find that the new volunteers do not aspire to be taken for nurses, and doubtless their wishes will be entirely respected. The essentially womanly profession of the trained nurse is not thought sufficiently heroic for the W.V.M.S.C. There is a slightly contemptuous ring in the remark, "These nursing sisters never go nearer the scene of action than the Base Hospital, and are naturally fitted by training only for nursing duties." And to "fitted by training" might well be added "fitted by nature" too. However, it is unlikely that many English girls will consider the making of wounds ranks higher than endeavours to heal them. We trust that in our own times at any rate the application of bandages will be more popular with our women-kind than stretcher drill or shoeing horses.

### A HIGH FEE.

Our readers, private nurses especially, would doubtless be glad to know where employers can be found willing to pay seven guineas a week for their services. That is the sum which is, so the correspondent of a Lancashire paper asserts, received by private institutions who farm out their ill-paid nurses. A paragraph similarly worded has appeared in other country journals, and the writer who comments on "stories that are afloat of a somewhat sensational character" would do well to get more accurate details as to nurses' ordinary salaries before attempting to instruct country readers. If private institutions still obtain nurses at £25 per annum willing to earn large fees for them, the fault lies with the employed. Good training and satisfactory certificates of moral character and efficiency can command fair fees for any woman, but seven guineas a week is a fee which in England at any rate exists almost exclusively in the imagination of the journalist.

### ST. BARTHOLOMEW'S PROBATIONERS.

There has been some correspondence in our contemporary, *The Lady*, on the regulations of a metropolitan training school, and regarding the justice of the code of rules various opinions appear to prevail. One of the nursing staff writes in last week's issue that "the rule relating to the payment or non-payment of probationers according to their conduct may, to the uninitiated, sound unkind or unjust." The point in question was forcibly dealt with in The Hospital of January 20th, under the heading "Work Without Wages." The "conduct" of probationers apparently includes not merely conscientious fulfilment of duty, but also the difficult task of giving "satisfaction" to the medical and surgical staff and heads of the nursing department. The current quarter's salary is also to be entirely withheld from "anyone who from any cause whatever leaves the hospital in the course of a quarter." *The Lady's* correspondent, who signs herself "A Present Sister," does not defend these rules which she thinks the "uninitiated" (*i.e.*, the unprejudiced outsider) may think "unkind," but she evidently considers they should not be attacked for their injustice because they are so seldom enforced. There is an illogical charm about this argument which speaks for itself. And so does the writer's frank avowal that salaries are paid for months at a time to nurses who are off duty.

### TESTIMONIALS.

"Have you no others?" asked one of the Selection Committee, laying down a sheaf of eulogistic testimonials. "I thought these would be sufficient," replied the amazed candidate. "Well, there are plenty of them, certainly, but my dear lady, they are all from doctors, and some of them from quite newly-qualified men." "I know they are," said the candidate, plucking up a little spirit; "they are always glad to help a nurse on." "Glad to hear it," rejoined the old gentleman, gruffly, "but one truthful recommendation from a practical matron or head nurse is better than a dozen of these flowery compositions. If you can get a recent one, from a lady, we will consider your application again." The crestfallen lady withdrew, doubting very much whether a matron, who knew her in her work, would be likely to describe her as the semi-angel those young men had called her after a short and superficial acquaintanceship.

### GENUINE APPRECIATION.

"I suppose there's a lot of rules to keep down there, father?" said the pale-faced boy who had just been offered a three weeks' visit to a convalescent home. "Sure to be, sonnie; there most generally is heaps of 'em at them places. But there's plenty to eat, and they'll treat you kind," rejoined the working man, consolingly. When he met his little son on his return from the sea, the boy, pale and delicate no longer, rushed at him eagerly: "Father, father, you was wrong! There ain't no rules at all. So long as you behaves decent nobody never says nothin', and there's meat every day, plenty! and a donkey cart to drive out in, and a bed to yourself, and a box for yer clothes; and, oh! father, don't I wish I wur just goin' back again."

### LEEDS LADIES' HOSPITAL FUND.

The example of the organisers of the Workpeople's Hospital Fund has been recently followed at Leeds by a number of ladies. A well-attended meeting took place on March 13th at the Town Hall, the Mayoress, Mrs. Leuty, being elected first president of the new association. Many supporters of the Workpeople's Hospital Fund were present, amongst others Alderman Sparks, who pointed out the marked success which had already attended their efforts. It was finally agreed that the name of Leeds Ladies' Hospital Fund should be given to the proposed organisation, and it was resolved, "That the ladies of Leeds at this meeting hereby determine to help the Leeds medical charities by frequent periodical collections from those classes who do not already subscribe to the funds." We wish all success to an effort which ought at least to bear as good results as those which have already attended the well-organised scheme of the Leeds working men.

### GOOD WORK AT HINCKLEY.

Hinckley Cottage Hospital continues to be well supported, and the third annual report tells of its steadily increasing usefulness. A probationer has been added to the staff, and a cottage to the hospital. The latter gives much-needed extra accommodation, and enables a room to be set apart for paying patients. The Matron, Miss Rodgers, is an excellent nurse, and the record of patients successfully tended after operations during the last year is certainly one upon which all concerned may well be congratulated. Miss Caldwell, the District Nurse, has done valuable work amongst the poor in their own homes, extra nurses being engaged during the epidemic of diphtheria.

### A NEW ISOLATION HOSPITAL.

A well-arranged infectious diseases hospital is in course of erection in connection with the Union Workhouse at Fishpool, Bolton. The accommodation for nurses and patients seems to have received adequate consideration and so has their complete isolation from the other buildings of the workhouse. The guardians appear unanimous in a determination to do the best they can in every way for the sick and aged poor in the Bolton Union.

### STIRLING.

The Stirling Sick Nursing Society has held its annual meeting and given a most favourable report of the work of the district nurses. As one of the speakers remarked: "People were now inclined to wonder how they could possibly have done so long without them." The record of the three years' work of the society is altogether satisfactory. It is to be hoped that the appeal for sufficient funds to support an additional nurse will receive a liberal response.

### QUEEN'S NURSES IN DUBLIN.

The work done by the district nurses of St. Patrick's Home, affiliated with the Queen's Jubilee Institute, has steadily increased. A superintendent, four staff nurses and five probationers, being fully occupied in attending to the calls made upon their services, and an additional nurse had to be borrowed from London. The emergency meeting, summoned by the Archbishop of Dublin, president of the Home, in December last, resulted in a sufficient sum being raised to free the association from debt. It is most desirable, therefore, that increased subscriptions be forthcoming during the present year for the adequate maintenance of such admirable work amongst the sick poor.

### SHORT ITEMS.

The April number of "Nursing Notes" contains an interesting article on heart disease by Dr. Ryle, and one on the Royal National Pension Fund for Nurses. —At the first annual meeting of the Bramley District Nursing Association a most satisfactory report was given of the work done by the two nurses.—The Berwick and District Ladies' Nursing Association has now two nurses, and its second annual report tells of much good work done; the association is affiliated with the Queen's Jubilee Institute.—The Kidderminster Guardians have voted grants to the District Nursing Associations in the following parishes: Wribbenhall, £10; Bewdley and Stourport £15 each.—The Guardians at Nottingham have subscribed £60, for one year, to the Notts and Nottingham Nursing Institution, which has undertaken, we learn from the local press, "to nurse the whole of the outdoor sick poor having relief from the Guardians."—The Woolton District Nursing Society has issued its fifteenth annual report, which is a very satisfactory one, and shows Nurse Key's work to have been thoroughly appreciated by the committee as well as by her patients.—The Leamington District Lady Nurses' Fund has a famous record of work, the second nurse, Mrs. Biddlecomb, holds the diploma of the London Obstetrical Society, and undertakes midwifery cases for the Provident Dispensary, in addition to assisting Miss Joyner, the head district nurse.

### On General Nursing.

By ROWLAND HUMPHREYS, M.R.C.S., L.R.C.P.Lond.

### V.—SCARLET FEVER—(*continued*).

### VARIETIES.

The chief varieties of the disease are :—

1. *The latent form*, sore throat occurring not unfrequently in other members of a household where there has been a case of scarlatina, and in these persons the mildness of the complaint neither prevents its complications nor its infectiousness.

2. *Surgical scarlet fever*, a variety which occurs in operation cases, is characterised by its mildness, as well as by the irregular appearance of the rash.

3. *The malignant form*, known by the severity of the symptoms from the outset, various complications appearing in its course if the patient survives the first few days. Alcoholic stimulants are of the greatest service in these cases, and should be reserved for them, as they have a tendency in milder forms to send the temperature up. The hyperpyrexia has to be treated by the cold pack, or bathing, or sponging; the typhoid symtoms by predigested food and cardiac stimulants, and the complications as they arise. Cancrum oris, sloughing of the extremities, &c., may occur in this disease, and others where the vitality has undergone depression.

4. *Scarlatina anginosa*, a form to which the severe ulceration of the throat gives its name.

5. Finally, the variety which attacks lying-in cases may be mentioned, if only to reassure those who have to do with such cases, or where the two cases coincide. There is no especial form of the disease peculiar to the puerperal state; and the author has on two occasions had to deal with a scarlet fever and a puerperal case without the mother in either case taking the disease, although in one of them the fever case was found in bed with the mother within a day or two after her confinement. Of course the strictest antiseptic precautions were taken, though, in one case, the child could not be removed from the house.

The treatment of scarlet fever has been sufficiently indicated already, in that it should be directed towards the prevention of complications. The main point, in a mild case, is to see that the disinfection of the patient is efficiently carried out. The patient should be strictly confined to bed for three weeks. During this period the skin should be sponged down twice daily, and then rubbed all over with camphorated oil, glycerine, vaseline, eucalyptus oil, carbolic oil (1-40), or other suitable application, and the application continued for six weeks with bathing twice a day when the patient is up. Disinfection of everything in the room, a separate set of table appointments, nothing entering the room being allowed to go down stairs again till after thorough disinfection is also necessary. The throat trouble may be much relieved by sucking ice, inhaling steam, using gargles, and antiseptic applications. Great care and attention must be exercised to prevent or at least to mitigate the spread of the inflammation to the internal ear. The nasal douche is of the greatest service where this has taken place, but the doctor will probably pass the Eustachian catheter, or use Politzer's inflater to keep the communication between ear and throat open and so prevent the purulent discharge being pent up in the cavity of the tympanum, and bursting through the drum destroy the ossicles or other delicate parts of the organ of hearing. If the condition is recognised early, and even if the drum is already perforated, free and frequent douching will often result in re-formation of the membrana tympani, and complete return of the hearing power. The great point is to give the discharges free vent. The throat may be disinfected by a gargle of salicylic acid; this will not only disinfect, but will assist the infectious particles of epithelium to separate. Diphtheria and scarlet fever seem to be associated together in some epidemics; they do not appear to influence one another in any especial way, except that the diphtheria often follows the other complaint, and so attacks a patient already weakened by previous illness. The nurse should wear a cotton dress and apron in the sick room, and this should never go outside it. If a sheet be hung over the door, and kept moist by some antiseptic solution, it serves as a warning to thoughtless people, if of no other good. The nurse's hands should be washed just before leaving the room, and dried outside the door on a towel kept for the purpose. All excreta should be mixed with some antiseptic at least an hour before they are thrown away, and if there is no drainage system, they should be buried in the ground some little distance from the house, and not thrown down the closet.

A few words may be added as to the practical disinfection of a room after an infectious case has occupied it. First the size of the room should be roughly estimated by multiplying together the height, breadth, and length, and then allowance should be made for any articles of furniture, projecting walls, &c. For each 100 cubic feet thus calculated, 1½ ounces of sulphur in lumps should be allowed, as the air has to be well saturated with the fumes. The sulphur is placed in a metal vessel (of any description almost) supported on a pair of tongs over a basin of water, some spirit is poured on to the sulphur, and set fire to. The room should previously have been prepared by sealing up the cracks on each side of the window frames with paper and paste, closing the register, and closing all apertures however small. The things to be disinfected are hung on lines across the room, the sulphur ignited, and the room kept hermetically closed for 24 hours. The windows are then opened, the walls stripped and washed, and the floor scrubbed with some antiseptic, and the walls then repapered.

The following table indicates the periods at which the important points in scarlet fever make their appearance, and shows the dates after the onset of the complaint between which the complications mentioned may be expected to make their appearance :—

| Day. | Usual Course. | Possible Complications. |
|---|---|---|
| 1 | Renal suppression, sore throat, fever, pulse rapid, cervical glands swollen. | Nephritis. |
| 1— 2 | Rash appears on neck. | |
| 3— 4 | Temperature at its maximum. | Œdema of glottis. |
| 3— 5 | Rash at its highest, temperature and pulse fallen. | Dangerous period. Severe ulceration of throat, nose, &c. Inflammation of cervical glands. |
| 5— 6 | Rash fades, tongue strips. | |
| 6—10 | Desquammation begins. | |
| 6—12 | | Rheumatic complications. |
| 8—14 | Temperature becomes normal. | General anasarca; acute nephritis; spreading ulceration of throat, nose, ear and neck affected. |
| 14—20 | | Anasarca, with fever. Nephritis, with or without fever. |
| 15 | Pulse normal. | |
| 20 | | Slow or irregular pulse, indicating probably nephritis. |

*3rd week.*—Desquammation may end.
*6th week.*—Desquammation usually finishes.
*8th week.*—Infection usually ceases, even though peeling has not.

*Sequelae.*—Tendency to nephritis, sore throat, and skin affections.

### The International Medical Congress at Rome.

The eleventh International Medical Congress commenced its meetings in Rome on the 28th inst. One of its promoters in Italy is Dr. L. Westenra Sambon, whose father is the well-known archæologist, J. Sambon. H mother is an Englishwoman, a cousin of the late Charles Dickens. Dr. Sambon commenced his medical studies at Naples, and continued them at St. Bartholomew's, devoting himself especially to the diseases of women under the direction of Matthews Duncan and Dr. Clement Godson, to whom he was clinical assistant, also assisting Sir Spencer Wells in operations. He received a gold medal from the Italian Government for his services during the cholera at Naples in 1884, he being then but nineteen years old; and in 1887 received another medal for similar services at Pozzuoli. In 1891 he attended, as delegate from Naples, the seventh International Congress of Hygiene and Demography, held in London, and read a paper on "Measures Adopted for the Prevention of Infectious Diseases and their Relation to our Knowledge of Epidemics." He was at the same time commissioned by the Italian War Office to study the organisation of the English Army Medical Service, on which he subsequently published a critical, but in the main complimentary book. He is also a frequent writer on natural history and phenomena. He is now settled in practice in Rome, where he has set his countrymen the wise example of employing English trained nurses instead of the ignorant though kind-hearted *sœurs de charité* generally employed as nurses. His recently-delivered lecture, in capital English, at the British and American Archæological Society in Rome, on "Medical Science amongst the Ancient Romans, as Deduced from Monuments," was a great success, and he has been asked to repeat it during the meeting of the Congress in April.

Dr. Sambon's interesting lecture commenced with a survey of the sanitary conditions of ancient Rome, which almost makes us who have had experience of its smells and fevers long for the return of the Cæsars. To hear of the Etruscan sewers, built 25 centuries ago and still in use, is tantalising to London householders, who are apt to be told by their plumber about once in two years that their "drains must come up" and be relaid and repaired. Very ancient also are the 14 famous aqueducts, mostly underground, which bring water to Rome from a distance of 359½ miles. Why, if these memorials of the sanitary science of Rome still exist in use have the sumptuous baths, one of which alone accommodated 3,200 bathers, ceased to be used by their descendants, with whom bathing is, unfortunately, not now a national custom? Food and clothing were also controlled by sanitary laws in the good old days when "Salus suprema lex esto" was a Roman maxim; for the Romans evidently believed that "prevention is better than cure," a point which the lecturer thinks is but now becoming the maxim of latter-day physicians. Free-born Romans were not at first inclined to become doctors, but they permitted their slaves to study under the Greek practitioners who settled in Rome, and many of the liberated slaves also qualified themselves to practice, receiving a licence from the Greeks after a short course of instruction and sharing their subsequent profits with their former teachers. Julius Cæsar, however, did his best to elevate the social status of the doctors by granting to all medical men the right to vote, and this induced many free citizens to adopt the profession. Celsus, author of a still famous medical treatise, was one of these. Specialists seem to have been as numerous as they are in our own time, and women doctors were also permitted to practice in medicine and obstetrics. Various ancient inscriptions referring to eye and ear specialists and their various instruments, and the seals affixed to their patent medicines, still exist. Dentists appear to have flourished, and six skulls were recently discovered in an old tomb with teeth fixed with gold as in the modern American bridge system. One of the false teeth was a horse's tooth cut down to fit the human mouth. Most advanced surgery appears to have been practised, for which the instruments were most ingenious. Trephining seems to have been successfully performed, as also operating for cataract, removal of stone from the bladder by suprapubic operation, and the Cæsarian operation to deliver a live child from the corpse of the mother, by which the lives of Cæsar and Scipio Africanus are said to have been saved.

The profession must have been well paid, for we read that Stertimus earned £6,500 a year, and the surgeon Canie received £2,000 for one operation; though cases occurred when patients who in extremes offered large sums for their cure withdrew their promises on recovery, and these could not be enforced by law. Votive offerings, which were virtually bribes offered to the Roman deities, were frequent, and might consist of land, jewels, animals, even rusty tools and cast-off clothing, crockery of all descriptions, and coins; these latter when now discovered near the ancient shrine, are useful in determining the period at which it was in vogue. To us the most interesting offerings are the terra-cotta statuettes of deities, human beings, animals and fruit, also portions of human anatomy, such as the stomach, liver, and small intestines, all placed in the middle line, and showing a curious conventional anatomy. This was because the Romans had an imperfect acquaintance with human anatomy, as they were forbidden to dissect dead human bodies. Galenus himself in his book teaches the anatomy of the monkey as equivalent to the human, and all the common domestic animals were studied for the same purpose. Dr. Sambon illustrated the lecture by exhibiting many specimens of votive offerings of all ages, and it is curious to note that in many remote Italian towns this superstition still exists, and is practised in spite of the many changes in creed and medical practice; and still more curious it is to note that latter day votive offerings resemble almost exactly in form and material those which were dedicated to the ancient gods.

### St. Thomas's Hospital Chapel.

### DEDICATION SERVICE.

On Easter Eve, in St. Thomas's Hospital Chapel, at a quarter to eight p.m., an altar and cross were dedicated to the glory of God, and in memory of Agnes Murray, a regular worshipper in St. Thomas's Hospital Chapel from 1880 to 1887. The altar of oak has been designed in the classic style of architecture in harmony with that of the chapel. The front is divided into three panels formed by fluted pilasters, moulded arches, and spandrels. Each panel contains a representative figure carved in bas-relief and decorated in appropriate colours on a recessed background of gold. The centre figure is that of our Blessed Lord as the Great Physician, robed in garments of red and blue, signifying heavenly love and heavenly truth. On His right stands St. Luke, depicted as Evangelist and physician, holding in his hand a rod and serpent, symbol of healing. On His left is St. Thomas the Apostle, the patron saint of the hospital, bearing his distinctive emblem, the builder's square. The altar has been designed and executed by Mr. J. Roddis, Aston Road, Birmingham. The cross, by Messrs. Hardman, Powell, and Co., King Edward's Road, Birmingham, has been designed in harmony with the chapel architecture. It is of polished brass, lacquered, with sacred monogram in centre executed in *repoussé* work, and bears an appropriate inscription.

### Women's Work.

### PRACTICAL MASSAGE.—I.

By a Masseuse.

In my article on "Massage in Java" I gave a glimpse of massage in its original form as still practised by the natives of India, China, and almost all the "islands." We English who are so forward in most things, or at least pride ourselves upon being so, which is not quite the same thing, have been very backward in taking up massage, in fact, behind almost every other European country in this matter. I suppose after all it is only the force of example which has compelled us to adopt the system. This is all the more extraordinary as "rubbing" pure and simple has been advocated from time immemorial for such complaints as rheumatism, sciatica, lumbago, chest affections, in fact, any ailment resulting from cold as well as weariness, and general aching of the bones. We now find it an excellent remedy for corpulency, emaciation, anemia, hysteria, debility, insomnia, neuralgia, stiffness of the joints, sprains, writer's cramp, &c., &c. Some years ago, when massage came to the fore, as it were, there was quite a rush to learn it, and "still they come," and the little body of masseuses are gradually swelling into an army of strong and active rubbers. Massage seems to agree with one somehow. But although the ranks are full almost to overflowing, this need not discourage a would-be recruit, for if we turn to any branch of woman's work at the present moment, aye, and some things which used to be exclusively men's work too, we shall hear the same old cry, "Overcrowded." I will assume I am speaking to those wishing to study the art of massage; they who feel they must make their way in the world somehow, and try their hands at something. First of all, I would ask have they considered the matter fairly and well, and taken all its bearings, so to speak? Do they, to put the question point blank, like rubbing? "One never knows what one likes without trying." True, in a measure; but yet in a matter like the point at issue it is possible to judge pretty correctly. Have the would-be masseuses ever had to rub the chest or shoulders of a sister or friend, and, if so, did they find the operation tedious and unpleasant, performed only as a duty to be got over as quickly as possible; or was it done with pleasure, as a matter of course, no two thoughts about it? A point to be considered is age. A girl should be quite four-and-twenty before she attempts learning massage. There are exceptions, of course, to every rule, but a girl ought to be something of a woman before she can hope to make a good nurse, and every masseuse should have knowledge of the art of nursing even though she may not be hospital-trained. Then a well-taught and thorough masseuse will have all sorts and conditions of cases to attend on. She will therefore need to be steady, prudent, and quiet in her demeanour, and scrupulously neat and tidy in her personal appearance; her carriage must be easy, and her manners pleasant, yet dignified; not awkward, self-conscious, flighty, or conceited. Above all she must be refined and pure-minded in dealing with her patients, male and female, able also to exercise tact, discernment and patience in ministering to their requirements. Without this no masseuse is likely to succeed. By "being quiet" do not imagine I imply dull; a bright manner is always pleasing, and a woman who knows how to go about her work in a methodical, confident, and comfortable way is sure to get on. Active and energetic she may be, but not rough; violence is often confounded with energy, and there could be no greater mistake. A masseuse must seek to keep a soft, warm, dry, and supple hand. A great deal depends upon the hand and the manner of manipulation, and though these qualifications are not all present, yet cultivation and perseverance will do wonders. What could be more unpleasant than a damp, cold, or flabby hand passing over one's body, its own moisture hindering the free, smooth action so essential to true massage. Instead of producing the dreamy, comforting sensation which soothes while it invigorates, massage would prove absolutely a source of irritation and discomfort.

*(To be continued.)*

### Notes from Melbourne.

(Communicated.)

The rate of typhoid continues high, and, as usual, follows the heat wave. For the week ending February 10th the number of cases for the whole colony was 100, with 14 deaths, the number claimed by the metropolitan area being 48, with 4 deaths. In the corresponding week of last year the cases for the whole colony numbered 70, with 6 deaths.

The Rev. William Christopher Bunning, who perished in the River Yarra on November 25th last, has, in his will just proved, left £100 to the Melbourne Hospital and £100 to the Hospital for Sick Children.

Dr. Stanley D. Reid has been appointed assistant resident medical officer at the Women's Hospital. There were four candidates for the position, the other three being lady M.B.'s.

At a recent fortnightly meeting of the Committee of the Melbourne Hospital, the recommendation of the honorary medical staff that an honorary anæsthetist should be appointed was adopted. Effect to the resolution has not yet been given, and meantime another death under chloroform has occurred at the hospital.

The Committee of the Austin Hospital for Incurables, who had last year, owing to the state of finances, reluctantly to close the wing for consumptives at that institution, have decided to reopen it early next month (March). It is the only medical refuge in the whole colony for pauper consumptives, and the want of it has created much suffering, although large numbers of patients are received into the various benevolent asylums. The Austin Hospital Committee have been encouraged to reopen their wards by the fact that there has been an increase in the monthly subscriptions. The maximum annual expenditure on the wing can be kept within the sum of £1,600. A special appeal was made at the end of the year, and an unknown donor came forward, under the name of "Sigma," with the offer of £50 on condition that £450 was collected by a given date. Money did not come in very freely and the time was extended a little, with the result that about £230 was subscribed; nevertheless, "Sigma," on the 5th inst., forwarded a cheque for £50, so that the Committee have something in hand for their reopened wards. The Austin Hospital does not receive so much support as it is entitled to for its excellent work. Not a few cases have occurred in which, after two or three years' rest and careful nursing, the patient has been able to return to everyday life and to earn his own living. This hospital possesses a cancer wing, where at present there are a few vacancies.

The Victorian and the Queensland Board of Health have both just advised their respective Governments that it is not desirable to enter the international convention (or agreement) as regards cholera, adopted last year at Dresden. The reasons given are: That Australia has been hitherto free from cholera, and unlike most of the countries signing the agreement the only mode by which the disease could be introduced is by sea. The Queensland Government is of opinion that under no circumstances should persons on board a cholera-infected ship be allowed to land until they have undergone a full period of quarantine. The difference between the well-guarded state of coasts and boundaries of European countries with their efficient and well-organised local sanitary authorities and the generally defenceless shores of Australia is strongly insisted upon. Both the Victorian and the Queensland Boards of Health are of opinion that the Australian Governments should act in concert in any action taken, but as yet nothing has been heard from the other Governments.

### Lectures at St. George's Hospital.

THE ANTISEPTIC SYSTEM—(*Continued*).

At the lecture on Friday, March 16th, Mr. Dent, with the assistance of Dr. Slater, gave his audience a glimpse into the world of micro-organisms by means of limelight slides. Healthy blood corpuscles were first shown, followed by specimens of various bacilli, the bacillus anthrax, the bacilli of cholera, croupo-pneumonia, typhoid, &c., while their almost miraculous powers of reproduction, the product of a single cell in twenty-fours reaching a number expressible in billions, was explained. People who have any wavering doubt as to the advisability of boiling all milk would be speedily satisfied as to the desirability of such a proceeding could they have seen the picture of a tuberculous cow which was thrown upon the screen, and realized that disease may be transferred to humans who drink the milk from such an animal in its natural state.

Erysipelas, pyæmia, and hospital gangrene are diseases which may start from wounds, therein being found a suitable soil for the ferment to multiply in. The surface skin is as a rule sufficient protection; but a wound, a scratch, a graze throws open the susceptible tissues, and at once affords the best possible soil in which poison may germinate. You cut your finger, the thing is done. If you look closely when the bleeding has ceased you will see tiny drops of a clear fluid, that is lympth, and here is the material for the germs to multiply in. It is sometimes said that cleanliness is all that is wanted, there is no need for antiseptics to destroy the germs; and absolutely perfect cleanliness would do, but that is not to be had in this world, any more than is absolutely clear air in London. The circulating blood in an open wound is all ready to gather these micro-organisms from the surrounding air. It is undoubtedly true that surgery practised on antiseptic lines is very much more successful than surgery practised on aseptic lines, the principle being this, that the dirt is there, it must be there, and we take measures to counteract it. Cleanliness is good but antiseptics are better, in London at any rate. In the country, with abundance of comparatively pure air, you may perhaps get on, using oxygen as a disinfectant, but in London and large towns antiseptics are absolutely necessary for the treatment of wounds.

### Antiseptic Agents.

Iodine was largely vaunted at one time, but it is but weak as an antiseptic agent. It is a very good remedy for ringworm. Some Irish children were once brought to St. George's Hospital for the treatment of this disease. They were rubbed with iodine, and then went back into the street, where, investigating each other's heads the iodine got into their eyes, and they set up at once a dismal howling, whereupon the story got about that there had been another case of cruel treatment at St. George's.

Mr. Dent enumerated the various other antiseptic agents in most common use, iodoform, the "fashionable" remedy just now, carbolic acid, boracic acid, &c., and gave an account of their uses, advantages, and disadvantages. Carbolic acid, he said, in the early days of its use might be noted for ruffling the temper even more than it ruffled the skin. Corrosive sublimate is by far the most powerful of these disinfecting agents.

### Ancient Antiseptic Surgery.

Some people think that antiseptic surgery is quite a new thing, and so it is in the manner in which it is now employed, but it does not only date from Sir Joseph Lister, though he and Pasteur have applied a flood of light to it. The Greeks and Romans were very accomplished medicine men. Among the Pompeiian relics have been dug up instruments very similar to those in use at the present day. When Julius Cæsar came over to England he was accompanied by a very fully equipped medical department. In those days surgery was largely performed with red-hot knives, a process not perhaps so painful as may be supposed. This was in order to prevent bleeding, and it no doubt also prevented the entrance of germs. One of the best examples of antiseptic surgery dates further back than the time of Julius Cæsar. If carbolic acid be poured into a wound, and it is then covered with wool so that the air is thoroughly filtered before it reaches the wound, the case has been treated antiseptically; and this was what was practically done by the Good Samaritan when he surgically attended the stranger and did what no medical man has done before or since, gave his patient a fee. He treated the case on perfectly antiseptic lines. Alcohol is a very good antiseptic, and nothing could have been better than the pouring in of oil, thus keeping out the air.

### Notes from South Africa.

By Our South African Correspondent.

The Medical Congress, held in Cape Town during the last week of December, should prove of lasting benefit to the advancement of medical science, social feeling, and sanitary matters in the Colony. Already we hear a Health Bill has been drafted, and will be presented to Parliament next session, and the subject of leprosy has engaged the careful attention, not only of the Congress, but also of a Special Commission appointed to investigate the subject.

It would appear that in this country leprosy takes on a different type to that prevalent in India, hence the conclusions of the Indian Commission can hardly be made applicable to the disease as met with here. For instance, Dr. Impey, the Superintendent of Robbin Island, who has under his care upwards of 600 lepers, maintains that in certain cases a "self-cure"—a rather unfortunate expression, by the way—takes place, and that these cases are no longer contagious or infectious, accordingly they should be set at liberty. It is to decide such questions and to arrive at some conclusion whether segregation should continue to be carried out that the Leprosy Commission was appointed. We await their decision with much interest.

Dr. Caldwell (Edinburgh) has been appointed Resident Medical Officer to the Albany General Hospital, Grahamstown, in succession to Dr. Truter, who leaves to commence private practice; and the Government have appointed Dr. Walter Adam (Rainhill Asylum) Assistant Medical Officer to the Asylum at Grahamstown.

Out here we look on with great interest at the progress that is being made to raise the status of the nursing community in England, and we note Dr. Harding, of Berrywood Asylum, is instituting a sort of guild for asylum nurses. In his efforts in this direction he has our entire sympathy, and we are sure if the South African Asylum Nurses are approached for their support it will be given most heartily.

We hear that many improvements are shortly to be effected at the Asylum, Grahamstown, and an effort is being made to commence the building of a new asylum on the mainland near Cape Town, under the superintendence of Dr. Dodds.

There is an outcry that the qualifications of a certificated nurse out here are made too easy, and an effort is being made to raise the status of the examinations to the level of those held in England.

### Wants and Workers.

Old linen will be very welcome to district nurses, by whom it is always urgently needed, as we have reminded our readers on various occasions.

### The Junius S. Morgan Benevolent Fund for Nurses.

Mr. Burdett, the Deputy-Chairman of the Royal National Pension Fund, in seconding the report which was presented last week, said: I have great pleasure in seconding the motion for the adoption of the report and statement of accounts, a motion which has been proposed to you in a speech containing facts which, I am sure, must have interested and, I believe, surprised you. I think the support which this Fund has received, and its present position of prosperity, must be as satisfactory to you as it is to the Council. (Hear, hear.) I am sure that its present position and prosperity are in a large measure due to the devotion and attention which our Chairman has given to the work, and which have made it possible for him to state those facts which he has given you in his speech. Now, Mr. Burns, our Chairman, has dealt mainly with the business of the Fund. This Fund, however, has two aspects; and I am charged with the pleasing duty of dealing with the other side—the Benevolent side. We have a Benevolent Fund, which was established in memory of the late Mr. Junius S. Morgan, than whom no one did more, in the early days of the Pension Fund, to make it the success which it now is. The work of the Benevolent Fund is in charge of Lady Rothschild and a committee. It entails a great amount of work—more than anyone not familiar with the details can well realise. It is perfectly true that the vast work of the Pension Fund itself is very heavy indeed; but the amount of correspondence connected with the Benevolent branch of it is only less onerous; and we must also remember, and feel deeply grateful, that all this onerous work I refer to is done for us by ladies without any expense or cost whatsoever.

The Benevolent Fund has been able to take up the cases of 58 nurses, who, if not for this Fund, would have been left in their hour of need without support or comfort. Of these 58 nurses, 23 are now in receipt of life pensions. They are mostly nurses who began work long before the days of the Pension Fund, who had no opportunity of placing their savings in a Fund like this, and so, when their working days are over, they find themselves without any definite support and succour, and therefore would necessarily have to go to the workhouse were it not for the Benevolent branch of this Fund. The Committee—I think very wisely—looking into all these cases, which they do with the utmost care, have laid down the rule that they will never help a case unless thoroughly. Thus, whether the case is one for temporary help or permanent pension, the Committee's policy is to look at the individual circumstances of every applicant, and to take care so to husband any little resources they may have as to enable them, with the grant given by the Benevolent Fund, to have an income at their disposal which will place them beyond any call or want in the future.

Now, this plan has resulted in one case in a grant of so large a sum as £50, given to one nurse. It is a remarkable case; it is mentioned in the report, and I may mention it here. It is the case of a nurse who had a succession of fever cases to attend to, and in the result her whole nervous system gave way, and she became paralysed and helpless. The doctor under whose direction this nurse had been working said her one chance of recovery lay in going for a prolonged sea voyage. She would have to go, with any prospect of benefit, say to the Cape and back, which would cost £50. Well, the Committee felt that £50 was a larger sum than they were quite justified in giving to one case, but they took the matter up and communicated with others interested in it, and a sum was raised which, together with a small grant from the Benevolent Fund, placed all that was necessary at the disposal of the nurse, who was sent out to the Cape, and returned at the expiration of several weeks perfectly restored to health, and able to pursue her calling as a nurse with pleasure to herself and satisfaction to the doctors and patients. She might not even have been alive to-day if it had not been for this Benevolent Fund. It is a matter for great gratitude and thankfulness that we have been able to have this Benevolent Fund as an adjunct to the business side of the Pension Fund, and by its means be able to do as good and useful a work as I have just mentioned.

Again, life pensions are not simply paid in deserving cases, and these cases then left to themselves. Whenever a nurse comes under the control of the Benevolent Fund she is visited regularly by the lady visitor. The members of the Committee sometimes take up a case themselves. And from the time we touch a poor nurse who is a suitable candidate for the Benevolent Fund we never lose sight of her; and all her circumstances are considered—her need and state of health—if she requires more than was given at first, additional help is afterwards given, and she is provided for in comfort for the rest of her days. Love is the basis of the work of the Benevolent Fund, and I think it is only right that we should have such a basis for this work, seeing it confines itself entirely to the succour of those whose lives are spent in succouring and helping the sick.

I cannot conclude without expressing, on behalf of the Council, and on your behalf, our great gratitude to Lady Rothschild for all the interest and time she has given, and to the other members of the Committee. We are especially indebted to the Lady Visitor, Miss Leigh. Although we have made her a grant, she herself, as feeling so interested in her work, has asked that it may be returned as a donation, and that she may be considered an honorary worker. Our warmest gratitude is due to Miss Pritchard, who has written hundreds of letters, and given up many evenings, in order that all cases should be thoroughly investigated and properly dealt with. I am sure your thanks, therefore, will be heartily given to those ladies.

There is another aspect of this work, which I think is a good one. It helps the hale and it helps the sick. The influence of this Fund, through the institutions and the nurses belonging to it, is now so extended that we never have a case that comes to us for relief from any part of the country, or even from the colonies, in which we cannot write to some one connected with the Fund to keep in touch with it, and report on it afterwards. That is its influence on individuals. It gets them to take an interest in the sick. It gets them interested in the whole Fund; and so it has been built up to such an extent that, whereas the income of the Benevolent Fund is small, still, after relieving all suitable cases coming to us, we have been able to increase the capital stock to the extent of something like ten per cent., or £1,000. (Hear, hear.) I think that is most satisfactory in every way. Finally the Benevolent Fund enables us to say that any nurses employed in this country, or in any part of the British Empire, who choose to become policy-holders, may rest assured, so long as they continue members of this Fund, that their material well-being will be absolutely provided for; and they can then pursue the work of their calling without any anxiety as to their own personal position when the time comes to give up work and to meet the misery and expense of sickness.

I have taken up rather more of your time than I intended. Still, we have not spoken much before about the Benevolent Fund, and I think it only right to bring out a few points connected with it, and specially recommend to you the report of the Benevolent Fund, given now for the first time with the report of the Pension Fund. I am quite sure the more the work is looked into the more it will attract and fix the attention of the public, who are

The Junius S. Morgan Benevolent Fund for Nurses—*continued.*

necessarily interested in nurses because we all must one day suffer from illness. This consideration should induce many people to look into this Fund, and to send further contributions for the Donation Bonus and Benevolent Funds, because in that way they may feel confident that the nurses can be benefited to the fullest extent—to a far greater extent than by merely giving gratuities to individual nurses.

Report for the Year 1893.

This fund was established in 1889, although the actual work did not commence until 1890. The organisation is now thoroughly consolidated, and the lines upon which the work is carried out have been tested and found satisfactory. Added to this the field of benefits has become so large and important that it is felt an annual statement of the work done is now called for. During the three years of the existence of the Benevolent Fund, fifty-eight nurses have received help in various ways. About twenty-two of these nurses may be regarded as life pensioners, and mostly consist of older nurses who, never having had an opportunity of joining the Pension Fund, would, without the assistance of the Benevolent Fund, be in a condition of destitution.

The next most numerous class of those who have received assistance consists of members of the Pension Fund, whom the stress of adverse circumstances has temporarily prevented from paying their premiums. Numbers of nurses have been saved from withdrawing from the Pension Fund, and thus losing a hold on its benefits by the vigilance of the Secretary of the Pension Fund, Mr. Dick, who refers all cases where help seems called for to the Benevolent Fund.

Lastly, the smallest proportion of recipients are those receiving grants for special causes, and allowances in times of sickness, where it has been shown that the nurse was unable to join the sickness branch of the Pension Fund.

Every case is most strictly investigated at the outset, and touch is kept with all annuitants and other beneficiaries of the Fund. Where it is possible, the nurses are visited from time to time by Miss Leigh, the lady visitor, such visits being most useful and much appreciated by the nurses. In the early years of the Fund it is very necessary not to overload the list of recipients, or to establish precedents from which it might be necessary to withdraw, as the field of work increases. Whilst no suitable applicant has been refused, the care that has been exercised has brought the Fund into a flourishing financial condition at the present time. Thus, whereas in 1891 the available funds stood at £10,493, they amounted to £11,061 on December 31st, 1893. Again, the grants during 1891 only amounted to £46, but in 1893 they were no less than £376.

The Committee are rejoiced to state that very few pensioners so far have been removed by death. On the present occasion they regret to have to record the loss of Nurse Lydia Beaty, a pensioner since 1891. She was the valued nurse of the Emperor Napoleon, and closed a useful life in comfort, owing to the help received from the Benevolent Fund.

The Committee think it will be interesting to quote one typical case in each class, to illustrate the work accomplished by the Benevolent Fund:—

Class 1. Life Pensioners.—Nurse L. B. was already nearly seventy years of age when the Pension Fund was established. After a life of hard work she had managed to collect £90, which she placed in the Fund to secure a small annuity. Becoming incapacitated from work, she made application for a little assistance from the Benevolent Fund. Her high character, the endeavours she had made to provide for herself in her old age, rendered her in the eyes of the Committee a suitable person to be added to the list of life pensioners, and she is now the grateful recipient of a pension which, with her savings, amounts to £26 per annum. In all cases the Committee arrange that every pensioner is comfortably provided for, and that no nurse shall be in receipt of a sum which, added to any income from other sources, shall amount to less than 10s. per week.

Class 2. Temporary Help in Sickness and Distress.—Help was asked of the Benevolent Fund for Nurse M., a policy holder of the Pension Fund, her spine having been injured whilst at a case, resulting in serious illness and subsequent disablement. Nurse M., a doctor's widow, was formerly a sister at a large London Hospital, and later the matron of a provincial institution. At the time of her application she was living with her sister-in-law, their united expenditure amounting to but 8s. weekly. The case was highly recommended. Nurse M. is still quite unable to resume work, but lives in fair comfort assisted by the Benevolent Fund to the extent of 10s. weekly. With part of this money Nurse M. pays her premiums, as she is most desirous of continuing a member of the Pension Fund.

Class 3. Special Cases. A Sea Voyage.—Nurse R. W., a policyholder, became totally disabled from work through a series of illnesses, culminating in spinal meningitis. Long ill-health had exhausted both the resources of the nurse and her friends, when one of the doctors for whom she had worked, and who was attending her, applied to the Benevolent Fund for aid to send her on a sea voyage, as the only likely means of restoring her to health. A voyage to the Cape, the shortest likely to prove beneficial, costs over £50. The Committee did not consider themselves justified in granting so large a sum, but through the energy of the deputy-chairman, the doctors for whom she had worked subscribed £10, Sir Donald Currie himself contributing one-quarter of the passage money towards the voyage, and the balance of £22 11s. 9d. was supplied by a grant from the Benevolent Fund. The result of the voyage on Nurse R. W.'s health exceeded the most sanguine expectations. On returning she visited a member of the Committee, presenting herself in robust health, ready and eager to resume work, which she has since continued to do, and full of gratitude to the Fund. Nurse R. W. has expressed her intention to return part of the grant when able to do so.

In reference to Rule (*b*) which heads this report, the Committee would point out that they have found it necessary to limit the number of annuitants elected in each year or two, and in all cases where grants are applied for, the needs of members of the Pension Fund have prior consideration.

Hitherto no special effort has been made to collect contributions, and up to the present time the Fund has been sufficient to meet the demands upon it. The work, however, is increasing so rapidly that unless subscribers are found the Fund may some day be unable to provide for the needs of the increased number of applicants. Those who have been benefited by the services of a nurse will, we hope, be induced to contribute to a Fund which aims at placing the necessitous members of the nursing body in a position of security and comfort. (Signed)

E. L. Rothschild, President.
Rosalind Pritchard, Hon. Secretary.

### Notes and Queries.

**Queries.**

(29) *Women Volunteers.*—Kindly tell me to whom to apply for further particulars regarding recruits for the new Women's Volunteer Medical Staff Corps.—*Enquirer.*

(30) *W.V.M.S.C.*—Where can I get a form for enrolment in the proposed corps?—*Infirmary Nurse.*

(31) *Sister's Post.*—Information wanted as to the appointments at Derby Hospital.—*Nesta.*

(32) *Nurses' Libraries.*—Where can I see a collection of all the principal nursing books?—*Staff Nurse.*

**Answers.**

(29) *Women Volunteers (Enquirer).*—The Hon. Secretary is Miss Ethel Stokes, and her address is 19, Euston Square, London.

(30) *W.V.M.S.C.—(Infirmary Nurse).*—See answer above.

(31) *Sister's Post (Nesta).*—Apply to the Matron for an answer to both questions.

(32) *Nurses' Libraries (Staff Nurse).*—Write to Scientific Press, 428, Strand, and ask for a catalogue.

☞ For Everybody's Opinion, Reading to the Sick, Book World for Women and Nurses, &c., see page cclix. et seq.

### The Muses' Looking Glass.

### IN RURAL DISTRICTS.—UNRECOGNISED PERILS.

"Now you will come, please, Mrs. Thompson?"

"Well, miss, I've brought up nine children, and I don't see what more I've to learn about them."

"Oh, it's not that you should learn, Mrs. Thompson, but we should so like you to hear the doctor, and give us your opinion on his lecture, you are such an authority on the subject, you see!"

"Oh, if you put it in that way, miss, I'm sure I'll be very pleased to come?"

It was to hear a lecture on the "Rearing and Treatment of Infants," to which the young lady invited Mrs. Thompson for the committee was most anxious to secure a large audience.

The day arrived, and with it Dr. N——, and Mrs. Thompson; the room was full, and the ladies' committee radiant.

The mother of nine seated herself well to the front and prepared to listen, and condemn or approve.

The lecturer introduced his subject in a masterly manner, speaking of the sweetness and delightfulness of babies; his audience felt at home with him at once; slight murmurs of approval showing he had struck the right chord, and he continued complacently, but with increasing warmth. "Who would willingly be cruel to the little angels? Not one of you here before me to-day! Yet it is possible that even kind-hearted women like you may be guilty, unintentionally, of cruelty." A little shiver of incredulity passed over the assembly; Mrs. Thompson frowned; this young man didn't know so much after all as she had begun to give him credit for. But he, unconscious of her criticism, went on to tell the audience how to bring up young children to be healthy. With this end in view, it is to be supposed, he gave a gruesome list of all the ills baby flesh is heir to. The young mothers shuddered; they would have enjoyed hearing about measles and whooping cough, and any such common complaints that they all expected their little ones to have, and would never feel thoroughly satisfied if they did not, but they were not prepared for this.

Dr. N—— distressed their tender hearts terribly by telling them how even they had caused their little ones to pass through the fire, how they made the bath a torture, and the cradle a misery; and as to feeding, well, most infants was either starved or shamelessly overfed. "How are we to prevent such things?" moaned one young woman in an agony of distress for her sweet babe at home, but it presently appeared that the doctor had gone back to what might be called ancient history, and was intent on Mrs. Gamp and her colleagues, declaring modern trained nurses to be reliable and altogether harming, and apparently growing everywhere thicker than blackberries, so that infants need never be sacrificed.

The audience looked a little suspicious, and thought this nice young man need not romance about the past to sensible nineteenth century women, but he suddenly regained their confidence by judiciously mentioning "croup." Here there was no doubt of his knowledge, "he really might have had croup himself, he spoke so feeling of it," suggested one old lady. He could tell all about the steam kettle, the temperature of the room, what should be done before the medical man arrives, and after he was gone, every little thing indeed that a detail loving woman could desire. But Mrs. Thompson looked sad as she realised that her babies must nearly have died when they had croup, and she had never known it. Things seemed getting serious, but the worst was yet to come.

Dr. N—— passed on to "Collapse." "It is a most serious thing," he said, "and if immediate measures are not taken, the baby may die even before the doctor can be called in. Now I don't as a rule care to prescribe stimulants for infants, but in such a case brandy is absolutely necessary."

Mrs. Thompson was perfectly aghast. She was an ardent teetotaller, and the idea of giving brandy to a baby was too awful; her imagination depicted an infant drinking brandy by the glassful, a confirmed little tippler, in fact a drunk and incapable baby. She could listen no longer to the lecture, which soon afterwards drew to a close.

"Wasn't it a good lecture, Mrs. Thompson; didn't you enjoy it?" queried Miss Jones, eagerly, as the audience dispersed.

"Well, miss, I won't say as it wasn't a good lecture, but don't you ever ask me to go to no more! I hadn't no idea of babies bein' so delicate; I've reared nine, and, thank Heaven, they're all as strong as needs be, but if ever I had to nurse another—well, I declare, miss, I couldn't do it. I'd be terrified the whole time that I'd let it fall, or scald it, or let it catch cold, or see it have croup or collapse or somethin'. No, miss, I shouldn't dare; why I'll scarce be able to look at a blessed baby again, let alone touch one."

Peter Parks.

### Royal British Nurses' Association.

### Its Finances.

Whatever may be the benefits which this association hopes at some future time to bestow on its members in return for their annual subscriptions, at the present moment its own special organ does not lay claim to much having been achieved.

"It has sometimes been said by members of the Royal British Nurses' Association that they receive no adequate return in the shape of privileges or advantages," says the *Nurses' Journal* in its last issue, "even for the small cost of their annual subscriptions," and, not attempting to deny the truth of this familiar speech, the "editorial" continues, "In considering the justice of their complaint it must be remembered that no association can confer advantages until it has lived long enough to be in possession of funds." It would be interesting to know at what age associations are usually supposed to find themselves possessed of funds, and from what source they are coming? At present the funds, we are told, would have amounted to £1,000, but, unfortunately "about that sum" has been consumed by certain law expenses.

Nurses may not all be keen women of business, but they are not likely to go on giving their support and subscriptions to any association which at the end of six years of existence can still only say, "We may now reasonably hope that every year will show an increasing balance of assets above liabilities, and the consequent gradual accumulation of a reserve available for the promotion of any objects which the members may have at heart."

### A Corps d'Élite.

"The Registered Nurses' Society" is the formal title of the new agency which, picturesquely described as a *corps d'élite* has been formulated by Mrs. Bedford Fenwick. It is stated to consist in an office for members of the R.B.N.A. only, and it promises that the nurses shall receive their own fees (as they do at other already existing institutions), less 7½ per cent. The only special feature announced in connection with this society is that the committee associated with Mrs. Bedford Fenwick is entirely composed of members of the association, from which the *corps d'élite* is to be drafted. No other trained nurses will be admitted. Hitherto these select nurses appear to have been somewhat undervalued, and "designedly kept in the background," but this can be no longer the case when a committee of registered members and "a paid superintendent" form a society for their special benefit.

### Discounted Fees.

The Sussex County Hospital is apparently the only hospital which has gone the length of openly promising a donation to probationers who join the British Nurses' Association at the end of their probationership, a portion of the fees paid for training being returned to each nurse who allows herself to be recruited after this novel and unique method.

### Everybody's Opinion.

### AN APPEAL TO THE BENEVOLENT.

Miss Harriet Middleton writes: Two years ago the Editor kindly inserted a paragraph in The Hospital, appealing for aid for a nurse. This was well responded to, and the nurse has been comfortably maintained ever since. From death and other causes, five of the subscriptions have recently lapsed, which makes a great difference in the small sum hitherto received regularly. I write to ask if you will allow the appeal to be repeated in The Hospital. Full particulars can be obtained from me by anyone who writes to "The Grange, Long Marston, Stratford-on-Avon." (The original appeal states that "a fully trained hospital monthly nurse, of eleven years proved usefulness, is now suffering from an internal complaint; has spent all her savings . . . . has neither relations nor means. A fellow nurse is anxious to give her a home, but has not the means to support her." To this appeal in The Hospital of February 6, 1892, the following note was appended: "We have enquired into the above case, and seen the certificate and testimonials of the nurse described.—Ed.")

### OLD TIME NURSES.

"Nurse K. G." writes: I hope the scheme for the Old Time Nurses has not fallen to the ground? I have spoken to many nurses about it during the last month and they all agree that it is much needed, especially now. There are many good and experienced nurses who, I am sure, would join. I think the motto "Tried and True" would be most suitable for the association. I would like to hear if any scheme is likely to be carried out as the nurses to whom I have spoken are often asking me about it. I hope soon to see something further about it in The Hospital.

### Appointments.

Macclesfield Infirmary.—Miss Sophia Wingfield has been made Matron of this Infirmary. She was trained at Addenbrooke's Hospital, Cambridge, and the General Lying-in Hospital, Lambeth, and afterwards held the appointment of matronat Ayr County Hospital. We wish Miss Wingfield every success in her new and important position.

Royal Hospital, Portsmouth.—Miss Grace Newbery has been appointed Matron of the Royal Hospital, Portsmouth. She was trained at the Nightingale School of St. Thomas's Hospital, where she afterwards held the post of ward sister. She was afterwards night superintendent at the Royal Infirmary, Liverpool, and subsequently assistant lady superintendent in the Nurses Training School. She takes with her many good wishes.

Chesterfield and North Derbyshire Hospital and Dispensary.—Miss Janet Lambton has been appointed Matron of this institution. She was trained at the Royal Infirmary, Wigan, was Head Nurse in charge of operating theatre at Salford Royal Hospital, Manchester; Matron of Rochdale Infirmary for five years, and Matron of Croydon General Hospital for four years. We congatulate Miss Lambton on her new appointment, and the committee on having secured a lady of such valuable and varied experience.

### Minor Appointments.

The Infirmary, East Dulwich Grove.—Miss Ellen Bracewell, "Sister" of one of the female wards, has been promoted to be Night Superintendent at this infirmary, and has our cordial good wishes in her responsible post.

Up-Country Nursing Association for Europeans in India.—Miss Amelia Preston, who was trained at the General Hospital, Bristol, has been selected as a nurse to this Association, and we heartily wish her a successful and useful career in India.

The Royal Infirmary, Liverpool.—Miss Fergusson, of St. Thomas's Hospital, has been made Assistant Lady Superintendent of the Nurses Home at the Royal Infirmary, and Miss Rigney has been appointed Assistant Lady Superintendent. Miss Rigney received her training in the institution, and was night superintendent there for some years. Miss Lumsden has now been made Night Superintendent. She, also, was trained and held the position of ward sister at the Royal Infirmary.

### For Reading to the Sick.

### CONTENTMENT.

**Motto.**

Content is health to the sick and riches to the poor.
—*Thomas Fuller*.

**Verses.**

I am satisfied,
I dare not ask; I know not what is best—
God hath already said what shall betide.—*Longfellow*.

Thou cam'st not to thy place by accident,
It is the very place God meant for thee;
And should'st thou there small scope for action see,
Do not for this give room to discontent;
Nor let the time thou owest to God be spent
In idly dreaming how thou mightest be,
In what concerns thy spiritual life, more free
From outward hindrance or impediment:
For presently this hindrance thou shalt find
That, without which all goodness were a task
So slight, that virtue never could grow strong!
And wouldst thou do *one* duty to His mind,
The Imposer's —— overburdened thou shalt ask,
And own thy need of grace to help, ere long.—*Anon*.

**Reading.**

It is not what we might be, surrounded by other influences under different circumstances, but what we are in the midst of either.—*Heart Musings*.

Let us seek earnestly for contentment . . . for He has given us all things richly to enjoy. Contentment is perfect rest and perfect peace; it asks for nothing, seeks for nothing, hopes for nothing but what God gives. . . . Contentment does not ask to see the reason why God does this or that. . . . With open hands it receives all His good gifts, and thanks Him for His love and care; it does not look onwards, knowing that God will provide; it has no wants, no cares, but to know Him more and to love Him better.

Do not seek to choose or change your circumstances, they are the best, the very best for you. "The only wise God" has chosen them for you, and that in true love; for "the Lord is very pitiful, and of tender mercy."

Neither seek to change the characters of those about you; for you are set amidst them for your discipline and correction. It is by no accident that those particular characters are brought into contact with you. Your Father placed them there. Their foibles, deficiencies, and mistakes are all for your profit. They are meant to supply the discipline which is gained in society by those in health. Many of the things which try us are meant most kindly. Always try to believe this, and receive each thing as such, unless there be plain proof to the contrary. . . . .

If any other lot would have been equally good for; if any other discipline would have taught you as much of the evil of your own heart, or of the love of God, depend upon it He would have given you the lighter and kept back the heavier. Do not argue with the tempter; do not let him persuade you that your circumstances are unsuited to you; but say at once, "Get thee behind me, Satan: for thou savourest not the things that be of God." God placed me here—it is the will of God. "God is love." . . .

The same answer will serve for every lot in life—for every trial:

"To wise hearts this certain hope is given
No mist that man may raise shall hide the eye of Heaven."

*Sickness, Its Trials and Blessings*.

### The Book World for Women and Nurses.

[We invite Correspondence, Criticism, Enquiries, and Notes on Books likely to interest Women and Nurses. Address, Editor, The Hospital (Nurses' Book World), 428, Strand, W.C.]

Maria, Countess of Saletto. Translated from the Italian of E. Arbib. (London: Digby, Long, and Co.)

It is a great pity the compiler of Maria, Countess of Saletto's history does not possess a greater sense of proportion as to the manner of its telling. The book is far too long, and its length detracts in a conspicuous way from the pleasure given by its perusal. The most interesting character in the narrative is that of the Dowager Countess Saletto, a modern among moderns. But our interest is also shared in the wife of an ex-officer of the army; in whose marriage a flaw is eventually discovered, which sets her free from a bad and tiresome husband in a remarkably happy manner; perhaps less often discoverable in real life than in the world of fiction. The book is written in an agreeable way, and can safely be placed in the hands of those who are as yet uninitiated in the evils of this world—that is, anyhow, more than can be said of Italian romances as a rule.

"Farewell, Love!" By Matilde Serao. (London: William Heinemann. 1894.)

Matilde Serao is no common writer; her conception of character, her artistic treatment of material, place her prominently among the first writers of Italy. Mr. Edward Gosse, in his introduction to this volume, points out much in Matilde Serao's career that is of interest to readers of her books. Her novels are masterpieces, and yet it is only the leisure hours of an editress of a popular Neapolitan paper which she can bestow upon them. "She still retains in her nature something of the news reporter's quicksilver," Mr. Gosse comments, "but it is sublimated by the genius of a poet." If there is realism in "Farewell, Love!" there is idealism too. The intensity and abandon of the heroine's love, unrequited as it is by the object of her affection, assumes, one might say, almost heroic proportions—a love whose force is at once Anna Acquavivea's strength and her weakness. The conception of a Southern brain, Anna's character may appear exaggerated, unreasonable, to the more Northern temperament. But Matilde Serao draws her with a consistency and pathos, treating her as the inspired victim of unrealized love and hope, which is not beyond a human probability. The book sustains its dramatic power throughout, and the conclusion, if tragic, is in keeping with what precedes it.

The Secret of Madame de Monluc. By the Authoress of "Mademoiselle Mori." (London: Methuen and Co. 1894.)

We are almost inclined to think that "Madame de Monluc" without the secret would have made still more attractive a narrative. For the very curiosity which the title arouses in our minds prompts the hasty reading of a book which would well bear the test of a more leisurely perusal. The particular charm of this authoress's writings lies more in the manner of her story-telling than in the story itself. In the instance of her latest contribution to literature this is perhaps especially evident. Here we find a plot, but narrowly within the confines of probability, atoned for by the delightful natural manner in which it is written. The character of Solange, grand-daughter of the Marquise de Monluc, is admirably drawn—her bright, childlike nature contrasted with the prison-like existence in the big Paris hotel. Destined to be immured in convent walls, one feels the passionate nature inherited from her Provençal ancestors will one day awaken to a more human outlet. Very prettily is that awakening described, when the romance which lay hidden in her girlish heart awakes. In the dreary monotony of her isolated existence, Solange comes suddenly and unexpectedly in contact with the young and distinguished Maxime de Langier; it is here where the story is a little improbable, and where Madame's secret plays too important a part. The course of Maxime's love-making does not run entirely through smooth waters, but has its reward in the end, and Solange is freed from the doom of weeping her fair eyes out in dark convent walls.

### THE MAGAZINES.

*The New Review.*—The Rev. Benjamin Waugh's article, "Our New Protectorate for Children," in the *New Review* should afford food for thought to the optimists who look upon education and teetotalism as the cures for all the ills that flesh is heir to. For Mr. Waugh has come to the conclusion that the tortures inflicted upon children by the sober, though less numerous, and at the same time less easily discovered than those inflicted by the intoxicated, are as a rule far more cruel, while among the better educated and better circumstanced he notes a spitefulness and refinement of cruelty which distinguish them unenviably from their poorer and more ignorant *confrères* in the art of child-baiting. It is against the members of these so-called "higher" classes, too, that there is the greatest difficulty in obtaining evidence in cases of cruelty to children. The artisan who lodges in one of a row of small dwellings is more or less open to public observation, and has plenty of neighbours who would be ready and willing to witness against him; but the same is not the case in larger and more isolated houses, where the only people who are really cognisant of the acts of the master and mistress are the servants, whose evidence, so says Mr. Waugh, judges and juries are very unwilling to accept. On the whole it appears that the people who illtreat their children the most are not the very dregs of the population; it is those who are in fairly comfortable circumstances—most of the society's convictions were those of men earning good wages—who seem to have most thoroughly imbibed the spirit of cruelty.

Professor Garner, in the *Pall Mall Magazine*, expatiates on the subject of chimpanzees and gorillas as he observed them during his three months' residence (partly in a cage) in West Africa. Many of the accepted notions concerning the gorilla, says the Professor, are mistaken; our pictures, for instance, which generally represent him standing on his hind legs, give us an entirely false idea of the animal's usual position and mode of progress, which is on all fours. Then we have been taught that gorillas, when angry, make use of sticks and stones to hurl at their enemies; this is quite wrong, they don't do anything of the kind. Neither, apparently, do they build themselves houses in the trees as rumour has related; at any rate, although Professor Garner offered a reward to any native who would show him one, he was never able to get a sight of any such thing. Gorilla stories, he says, are like ghost stories, always being told on the authority of some person or persons unknown. Lord Wolseley finishes "The Decline and Fall of Napoleon," treating his subject from the campaign of 1813 onwards. He attributes the Emperor's rapid descent from the pinnacle of power to the fact that "he was not the same man" in 1813 as in 1796 and 1805. It would surely be at least as correct to say that he had not the same resources or material to work upon. The French troops who went through the campaigns of 1813 and 1814 were neither the patriotic enthusiasts of Arcola nor the veterans of Austerlitz, but for the most part hastily levied boys, hurried to the front to take the place of the shattered and dispersed Grande Armée. Even with an army of such inferior calibre, and outnumbered at all points, he all but wrested victory from the allies; and had a vigorous, instead of an exhausted, France been at his back, he would, in all human probability, have repeated the successes of Italy and Austria on the plains of Champagne. It was as a statesman rather than as a soldier that he blundered; and Lord Wolseley accounts for the infatuation which led him to refuse the chance of fairly favourable terms from the allies by his arrogant belief in the ultimate predominance of his star.

